# Brain Networks Differ According to Levels of Interference in Spatiotemporal Processing

**DOI:** 10.1002/hipo.70011

**Published:** 2025-03-24

**Authors:** Rochele Castelo‐Branco, Ana Paula de Castro de Araujo, Karen Cristina Pugliane, Luiz Eduardo Mateus Brandão, Ramón Hypolito Lima, Hindiael Belchior, Ywlliane da Silva Rodrigues da Meurer, Arthur Antunes Pereira‐Costa, Flávio Freitas Barbosa

**Affiliations:** ^1^ Memory and Cognition Studies Laboratory, Department of Psychology Federal University of Paraíba João Pessoa Paraíba Brazil; ^2^ Department of Medical Sciences; Transplantation and Regenerative Medicine Uppsala University Uppsala Sweden; ^3^ Post Graduation Program in Neuroengineering, Santos Dumont Institute Edmond and Lily Safra International Institute of Neuroscience Macaíba Rio Grande do Norte Brazil; ^4^ Department of Physical Education Federal University of Rio Grande Do Norte Natal Rio Grande do Norte Brazil

**Keywords:** brain networks, c‐Fos, episodic memory, rats, spatiotemporal processing

## Abstract

The ability to form different neural representations for similar inputs is a central process of episodic memory. Although the dorsal dentate gyrus and CA3 have been indicated as important in this phenomenon, the neuronal circuits underlying spatiotemporal memory processing with different levels of spatial similarity are still elusive. In this study, we measured the expression of the immediate early gene c‐Fos to evaluate brain areas activated when rats recalled the temporal order of object locations in a task, with either high or low levels of spatial interference. Animals showed spatiotemporal memory in both conditions once they spent more time exploring the older object locations relative to the more recent ones. We found no difference in the levels of c‐Fos expression between high and low spatial interference. However, the levels of c‐Fos expression in CA2 positively correlated with the discrimination index in the low spatial interference condition. More importantly, functional network connectivity analysis revealed a wider and more interconnected neuronal circuit in conditions of high than in low spatial interference. Our study advances the understanding of brain networks recruited in episodic memory with different degrees of spatial similarity.

## 1 Introduction

Episodic memory is our capacity to encode and recall when and where a specific event (what) happened. It has been demonstrated that the hippocampus and other temporal brain areas are involved in this type of memory (Eichenbaum et al. 2012; Chao et al. 2020; Barbosa and Silva 2018; Drieskens et al. 2017). When two or more memories for very similar events are formed, however, they need to be disambiguated based on their specific spatiotemporal and sensorial features, a process called pattern separation (Hainmueller and Bartos 2020; Scharfman 2016; Leutgeb et al. 2007). Along with the capacity to form different representations for similar experiences, the brain is also able to retrieve information based on partial or incomplete inputs through a mechanism called pattern completion (Kesner and Hopkins 2006). It is thought that at least two hippocampal subregions are fundamental to these processes: the dentate gyrus (DG) and CA3 (Hunsaker and Kesner 2013).

Hunsaker et al. (2008) developed a spontaneous object recognition memory task that can simultaneously assess temporal and spatial aspects. On this temporal ordering for spatial locations task, rats can discriminate the order of stimulus appearance as well as their locations. Moreover, they developed a version of the task with low (LI) and high spatial interference (HI). The rationale is that higher spatial interference demands a higher pattern separation processing. They found that permanent lesions on dorsal DG impaired animals in the high interference condition, but not in low interference. This result suggests a specific involvement of DG in the processing of high loads of spatial pattern separation. In contrast, CA3 lesion animals were not able to discriminate locations in any interference conditions, indicating a more general role in the spatiotemporal processing of memories.

Even though lesion studies are suitable to evaluate a causal relation between a brain region and a behavioral output, they do not assess the dynamics of neuronal circuits underlying a task. To address this aspect, the expression of immediate early genes (IEGs) has been widely used to evaluate neuronal activity (Barbosa and Silva 2018; Mahringer et al. 2019) in many different behavioral tasks, such as operant conditioning (Bertaina‐Anglade et al. 2000; Svarnik et al. 2015), novelty‐preference paradigms (Aggleton and Brown 2005; Mendez et al. 2015), spatial training (Guzowski et al. 2001; Centofante et al. 2023), and episodic‐like memory (Barbosa et al. 2013; Auguste et al. 2023).

More recently, functional network connectivity analysis has been used as an important tool to understand how widespread neuronal circuits support different behavioral tasks (Terstege and Epp 2023). Therefore, we hypothesized that different brain networks are activated when animals process the spatiotemporal features of objects in a recognition memory task involving conditions of high and low spatial interference, that is, different degrees of memory similarity. For this purpose, we used a temporal ordering for spatial locations task, based on the protocol developed by Hunsaker et al. (2008), and also measured c‐Fos protein labeling to assess neuronal activity in the dorsal hippocampus and other temporal lobe areas, specifically the perirhinal cortex and the lateral entorhinal cortex, once both are involved in object recognition and object‐location association (Chao et al. 2020; Kuruvilla et al. 2020). Interestingly, we detected wider and more connected brain networks during conditions of high spatial interference when compared to low interference.

## 2 Materials and Methods

### 2.1 Animals

We used 16 male Wistar rats, 3 months old, weighing on average 350 g from the Dr. Thomas George Animal House at the Federal University of Paraíba. They were housed in groups of a maximum of four animals in standard plastic cages (30 × 20 × 19 cm) on a 12/12 h light–dark cycle (lights on at 6 am), with food and water ad libitum. All the procedures were carried out in the light phase of the cycle. Experiments were approved by the Ethics Committee on the Use of Animals (CEUA/UFPB, permit n° 04/2016) and in accordance with the guidelines of Brazilian legislation for the use of animals in research (Arouca Law, n° 11.794/08).

### 2.2 Experimental Protocol

The animals underwent a daily 30‐min acclimatization session in the experimental room before starting any procedure. For 3 days, they underwent a 10‐min habituation session in the open field. For five consecutive days, the rats were handled for 10 min to reduce stress related to the presence and physical contact with the experimenters. The object recognition task was performed in an all‐black circular open field (walls 45 cm height, 90 cm diameter), with proximal and distal cues placed at the arena walls and at the walls of the experimental room, respectively. Two copies of five types of objects were randomly used in the experimental sessions. The objects were made of plastic and had differences in height, width, shape, texture, and color. Each of them had their interiors filled with plaster to add weight and ensure that they would not be displaced during the task. All objects, along with the field, were sanitized with a 70% alcohol solution at the end of each day of the experiment and with a 5% alcohol solution between sessions to avoid olfactory cues for the animals. The arrangement of objects in the open field was pseudo‐randomized and followed the order of the behavioral protocol for the task.

### 2.3 Spatiotemporal Memory Task With High and Low Interference

**FIGURE 1:**
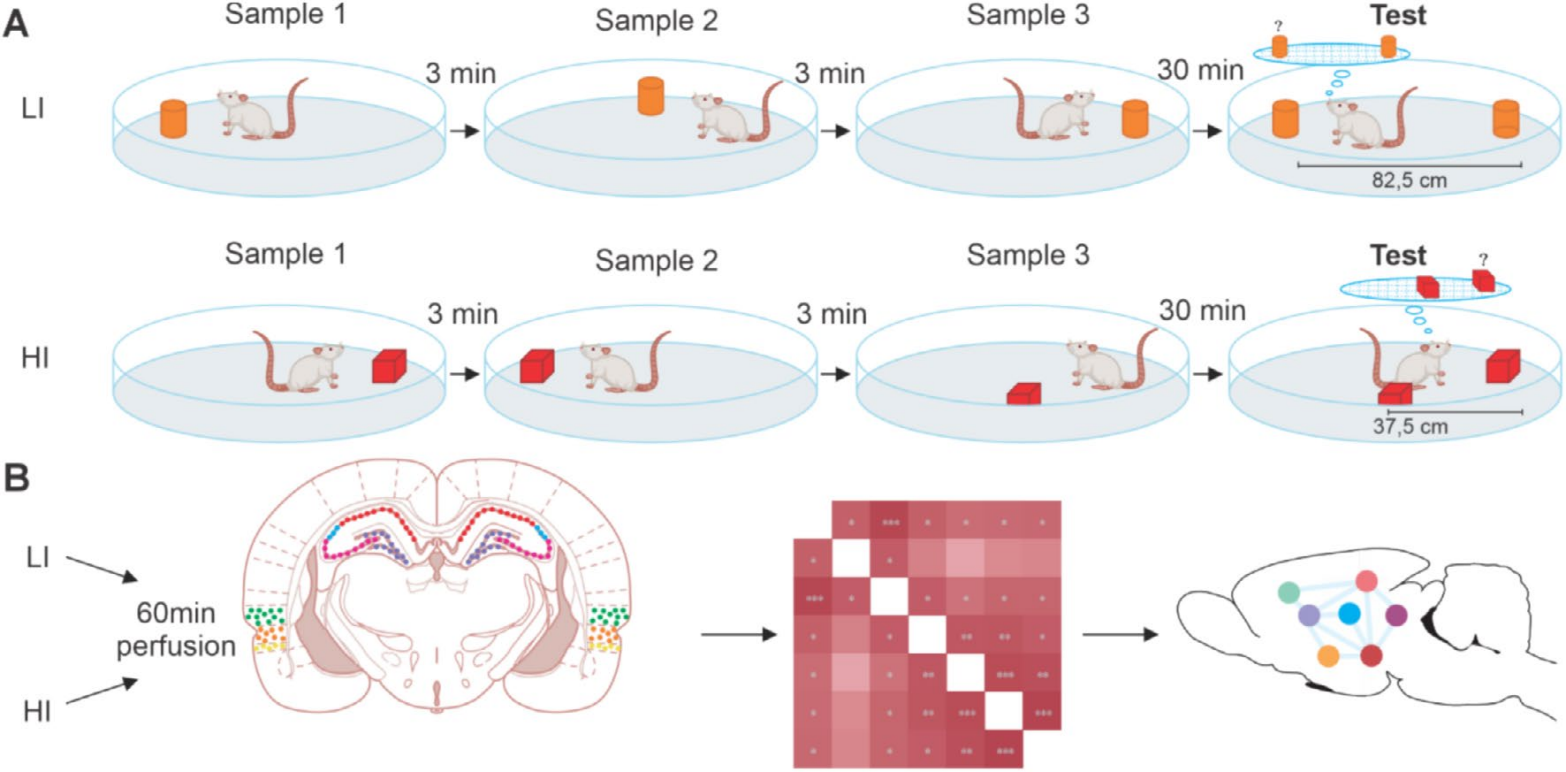
Protocol and experimental design for behavioral and immunohistochemical data. (A) Rats performed a spatiotemporal task with different degrees of difficulty. (B) Evaluation of neural areas recruited in each condition through c‐Fos expression, with posterior development of interregional correlation matrices. We investigated the perirhinal cortex (PRH), dorsolateral entorhinal cortex (DLENT), and the hippocampal subareas: DG, CA3, CA2, and CA1.

The behavioral task used here was based on the protocol developed by Hunsaker et al. (2008), which aims to assess spatiotemporal memory. The task has three sample sessions lasting 5 min, with a 3‐min delay between them. In each sample, an object is presented in different locations of the circular arena. After a 30‐min interval, a 5‐min test session was carried out, which was conducted in two configurations: low spatial interference (LI) and high spatial interference (HI). In the LI test, two objects are positioned in the arena, one occupying the spatial position used in the first sample and the other in the spatial position of the third sample, being separated by a distance of 82.5 cm, as illustrated in Figure 1A. In the HI test, objects are also positioned in the field in such a way that one object occupies the location of the first sample and another occupies the spatial location of the third sample; thus, the distance between the two is 37.5 cm, as exemplified in Figure 1A.

### 2.4 Perfusion and Immunohistochemistry For c‐Fos Quantification

The quantification of c‐Fos, a marker of neuronal activation, through immunohistochemistry (IHC) involves several key steps, including perfusion, brain sectioning, and the immunolabeling process. 1 h after carrying out the behavioral procedures, the animals were anesthetized by sodium thiopental administration (50 mg/kg, i.p.) and then transcardially perfused with 200 mL of heparinized saline solution (0.9% NaCl) followed by 200 mL of cold fixative 4% paraformaldehyde (PFA) in phosphate buffer solution (PBS, 0.1 M) up to ready for brain removal. The brains were postfixed in a 4% PFA plus PBS 0.1 M with 20% sucrose for 3 days at 4°C before being embedded in a cryoprotectant solution (PBS‐30% sucrose) for posterior sectioning by cryostat (Leica, Germany), with each section being 50 μm thick for analysis of the dorsal hippocampus and adjacent areas (Barbosa et al. 2013). All sections were stored in antifreeze solution; floating sections were incubated for 18 h with a primary rabbit monoclonal antibody c‐Fos (Oncogene Science, Cambridge, UK; 1:1000), containing 2% normal goat serum (Sigma, MO, USA), diluted in PBS 0.1 M, pH 7.4 plus 0.03% Triton X‐100 (ICN Biomedicals). Sections were incubated with a biotinylated goat anti‐rabbit secondary antibody (1:1000; Jackson Laboratories, USA) in PBS 0.1 M, pH 7.4 plus 0.0.3% Triton X‐100, pH 7.4 for 2 h at room temperature. Shortly thereafter, sections were washed and incubated in a 2% avidin‐biotin‐peroxidase solution (ABC Elite kit, Vector Labs, CA, USA) for 2 h. The reaction was developed by adding 2.5% diaminobenzidine tetrahydrochloride (Sigma, MO, USA) and 0.01% H_2_O_2_ in PBS 0.1 M, pH 7.4. The sections were washed four times (5 min) in PBS 0.1 M, pH 7.4 and, thereafter, dried, dehydrated in a graded series of alcohol, cleared in xylene, and covered with Entellan (Merck, Darmstadt, DE). The evaluation of immunohistochemical results was carried out with the aid of an optical microscope (OLYMPUS, BX‐41). The regions of interest quantified were perirhinal cortex (PRH), dorsolateral entorhinal cortex (DLENT), DG, CA3, CA2, and CA1, corresponding, respectively, to plates 64–66 and plates 72–75 in Paxinos and Watson (2013); see Figure 1B. Cell counts were performed manually in three sections per animal, using Image J software (1.46i, NIH), and the average count was calculated and used in the analyses. The experimenter was blinded to the experimental groups during counting. The mean values of each group normalized the number of cells for each brain area: (area value/average of LI and HI groups values) * 100 (according to Olarte‐Sánchez et al. 2014).

### 2.5 Behavioral Analysis and Statistics

The behavioral sessions were video recorded and analyzed using the Ethowatcher software (Junior et al. 2012) by a researcher blinded to the experimental manipulations: the level of spatial interference (HI vs. LI) and the label of the objects (first or third sample). We considered object exploration time intervals when a rat is faced toward an object at least 2 cm away from its snout. Differences were considered statistically significant with a *p* value < 0.05. Statistical procedures were carried out using R Statistical Software (RStudio, v. 4.3.1). We applied the Shapiro–Wilk test to analyze data normality in behavioral and IHC datasets. For behavioral analyses, a one‐sample t‐test was used to compare the discrimination indexes (D2) against chance levels (i.e., no preference: 0). To test temporal order recognition memory, we used a discrimination index that evaluates the animal's spontaneous preference for one of the objects. The discrimination index (D2) measure was calculated as the ratio between the time spent exploring the object positioned in the first spatial location (i.e., sample 1) (tOO) minus the object positioned in the more recent spatial location (i.e., sample 3) (tRO) and the sum of the time spent exploring both objects (tOO + tRO). Therefore, positive values for the DI represent a greater exploration of the first location over the last and indicate memory. For immunohistochemical data, a series of Mann–Whitney tests were performed comparing the two groups for each brain area analyzed. The “cor” function was used to evaluate Spearman's rank order correlation (rho) between the discrimination index and the c‐Fos expression.

### 2.6 Graph Theoretical Analysis

Graph theory is used to pinpoint central nodes and understand the information flow within a network. A key concept in this field is coactivation, which refers to a significant correlation between two active areas. Utilizing these correlations, graph‐theoretic measures can be applied to IEG data. Brain networks were developed from a symmetrical directed adjacency matrix, based on significant positive and negative Spearman's rank order correlations (*p* < 0.05) (Bullmore and Sporns 2009; Rubinov and Sporns 2011) in the levels of c‐Fos expression among pairs of brain areas, as shown in Figure 1B. To facilitate this, we used the Rstudio software with the “Corplot” package. Nodes represented brain areas, and their size indicated degree. All significant c‐Fos correlations between two brain areas were represented as edges or connections between nodes. The thickness of each edge corresponded to the strength of the correlation.

For graph analysis, we utilized Rstudio, integrating the igraph package and custom routines developed by our laboratory team (LEMCOG, UFPB, PB, Brazil). We calculated three centrality measures for each node: degree, strength, and betweenness centrality. The degree indicated the number of brain regions co‐activated with a given node. Node strength was the sum of all significant covariations in a particular area, with higher values denoting greater centrality within the network. Betweenness centrality measured how often a node acted as an intermediary on the shortest paths between other nodes, reflecting the brain region's intermediary role in network communication. Unlike static metrics like degree and strength, betweenness centrality captured the dynamic importance of nodes in facilitating network communication (Sporns 2018). The betweenness value was depicted by the node's color; warmer colors indicated a higher betweenness value (Auguste et al. 2023).

Another goal of graph theory is to assess the efficiency of the entire network. To achieve this, we calculated the global clustering coefficient and global efficiency. The global clustering coefficient assessed network segregation by determining the likelihood that two nodes, sharing a common neighbor, are directly connected. Global efficiency measures the network's integration, indicating the speed of information exchange within the network. For statistical comparisons between groups, we employed bootstrap analysis, drawing 100 samples from the original correlation matrix. The random model was generated by shuffling the rows and columns of the correlation matrix, preserving the main diagonal so each structure remained unchanged in the random model (Durieux et al. 2022).

The modules within the network were computed using the Louvain method. The Louvain resolution value was selected from the best value, representing the number of communities that repeat most in the two interference conditions, over the 100 random network repetitions. All statistical analyses were performed using bootstrap analyses. The differences were considered significant for *p* < 0.05. In order to measure the relevance of individual nodes within the network and obtain information about how information flows between them, bootstrap statistics were calculated on three centrality measures: degree, betweenness, and strength. The nodes were ranked according to each metric to reveal their relative importance in the respective network.

## 3 Results

### 3.1 c‐Fos Expression Does Not Differ Between Conditions of High and Low Spatial Interference

**FIGURE 2:**
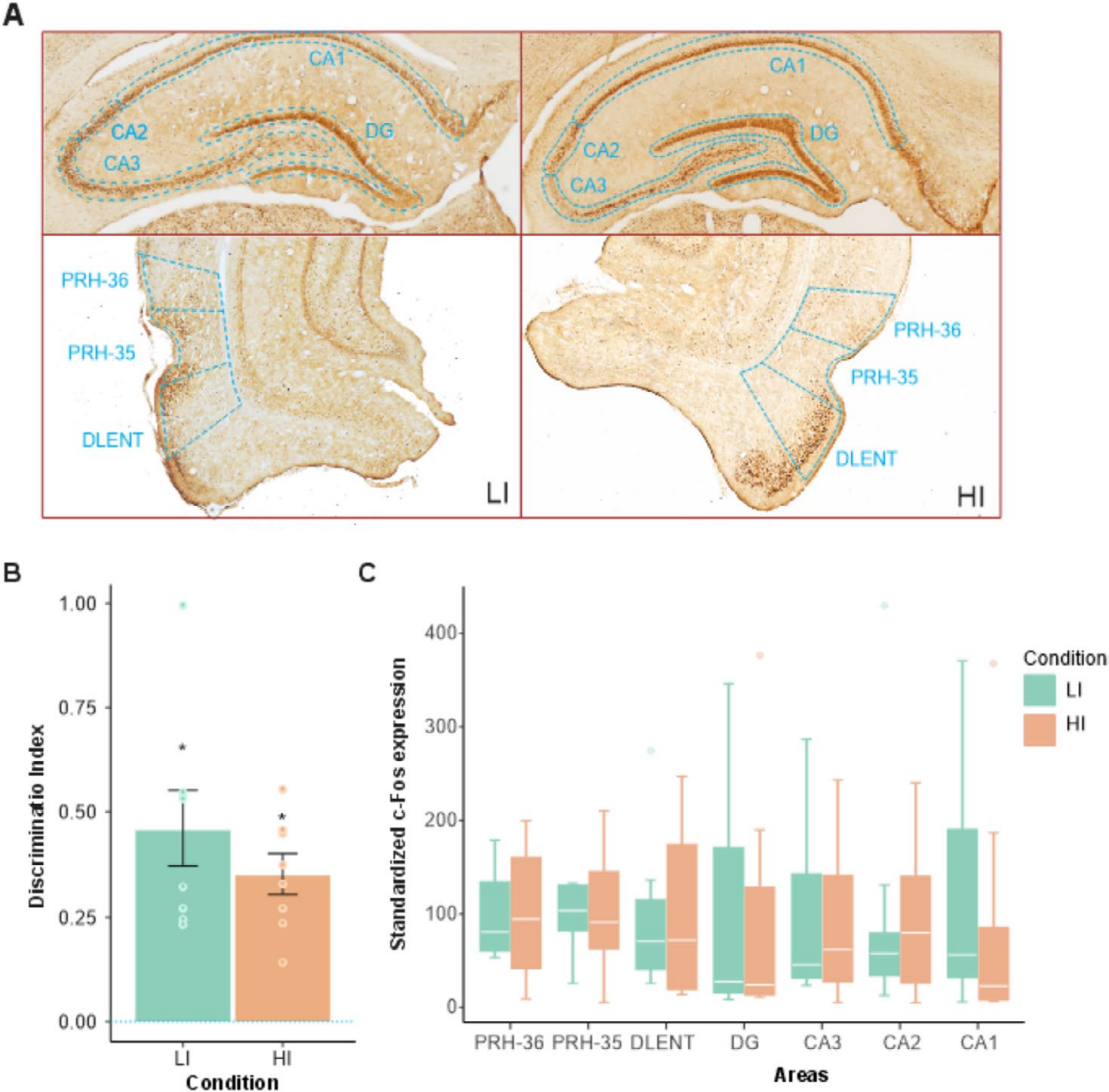
(A) Representative images of c‐Fos expression and delimitation of areas of interest for cell counting in the Low Interference Group (LI) and High Interference Group (HI). (B) Discrimination index (D2) for the LI and HI groups. The dotted blue line shows the value of chance. (C) Normalized c‐Fos expression in areas of interest, for each experimental group. *significant values above chance.

Animals did perform a task with high and low interference, and no rats were excluded due to a lack of exploration throughout the experiment (see Figure 1A). During the tests, the total exploration time in the LI and HI conditions was not statistically different, suggesting a similar motivational drive to object exploration during testing. A direct comparison of the discrimination index in LI and HI conditions revealed no significant difference (t(14) = 0,697; *p* = 0,49, d' = 1.525). The one‐sample *t*‐test found that both groups achieved discrimination indexes above chance, LI (t(7) = 4, 54; *p* = 0,003, d' = 5.11) and HI (t(7) = 9, 44; *p* < 0,001, d' = 7.29); see Figure 1D. This indicates that rats preferred the oldest object location over the newer one, indicating spatiotemporal memory, as rats naturally explore earlier items compared to recent ones. It is worth mentioning that they could discriminate locations in both conditions of spatial interference used here (82.5 and 37.5 cm) (Figure 2).

To assess the difference in c‐Fos expression between LI and HI conditions, a series of Mann–Whitney tests was performed comparing the normalized c‐Fos expression in the two groups for each brain area analyzed. However, no significant difference was detected (*p* values > 0.24; see Figure 2A,C) and (Table S3).

### 3.2 c‐Fos Expression in CA2 Correlates With Behavior Under Conditions of Low Spatial Interference

Spearman's rank‐order correlation analysis between D2 and c‐Fos expression showed a strong positive correlation in the CA2 region and the discrimination index for the LI group (rho = 0.78; *p* = 0.02). No significant correlations were observed for either the LI or HI groups regarding other neural areas (Table S4 shows the results for all correlations performed).

## 4 The Hippocampal Network Becomes More Integrated as Spatial Interference Increases

**FIGURE 3:**
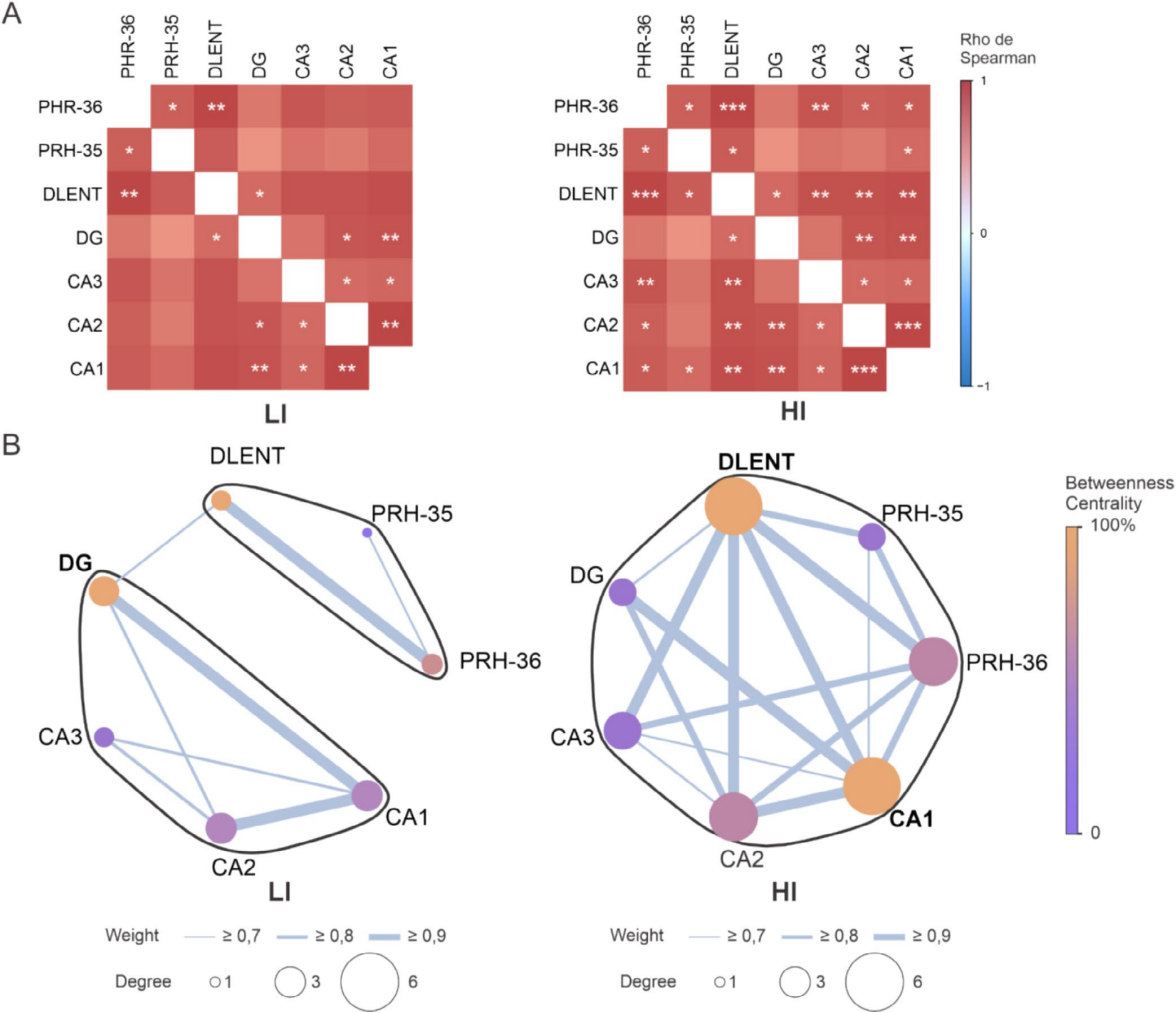
Network analysis of hippocampal and parahippocampal regions under different spatial similarity conditions. (A) Correlation matrix constructed from Spearman correlation between hippocampal and parahippocampal areas for the experimental groups (LI‐HI). Warm colors represent greater strength of positive correlation, while cold colors represent greater strength of negative correlation. * Significant correlations at *p* < 0.05. ** Significant correlations at *p* < 0.01. *** Significant correlations at *p* < 0.001. *n* = 16. (B) Graphs constructed for each group. Each circle corresponds to a node (region). The edges represent the correlation between the areas. The thickness of the edges represents the strength of the correlation. The colors of the nodes represent the level of centrality between them. The size of the node represents the degree. Black circles around a set of nodes represent individual clusters in the graph. The hubs in each network are depicted in bold.

To verify how the dorsal hippocampal regions interact as a network in each condition, we performed a graph theoretical analysis (see Figures 1B and 3). The LI and HI conditions presented 8 and 16 significant positive correlations, respectively, with different patterns of coactivation. For the LI condition, an interconnection between the dorsal hippocampal subregions (CA1, CA2, CA3, and DG) is observed, as it is among parahippocampal areas (PHR35, PHR36, and DLENT), but the network is weakly linked, with only one correlation connecting the parahippocampal regions with the hippocampus, through DLENT to DG (Figure 3B). In the HI condition, there is an intricate network with interconnections not only within the hippocampus and parahippocampal regions separately but also between them, where DLENT connects to the entire hippocampus, and CA1 connects to all parahippocampal areas, highlighting these two regions not only as intermediates between other regions but also in their number and strength of correlations (Figure 3A,B).

Network analysis revealed that for the LI condition, DG is central to the network. For the HI condition, the CA1 and DLENT regions can be considered hubs (see Figure 3 and Table S5).

The global clustering coefficient analyses showed that the LI functional network was dense and differed from chance (*p* < 0.001). In the global efficiency analysis, the HI network appeared robust and above chance (*p* < 0.001), suggesting that the networks were efficient in information processing and not segregated (Tables S7 and S8). Furthermore, the Wilcoxon test revealed a significant difference in clustering and global efficiency between both conditions, with the HI condition having the greatest clustering and efficiency, suggesting that the condition with the greatest interference requires a more robust network for information processing (Table S7).

Finally, the Louvain cluster analysis found different organizational modules for LI and HI conditions. For the LI condition, the presence of two clusters was observed, one formed by parahippocampal regions (PRH35, PRH36, and DLENT) and the other by hippocampal regions (CA1, CA2, CA3, and DG). The HI condition presented a single cluster in which all regions are integrated, as shown in Table S8. These results indicate that as spatial interference increases, the hippocampal and parahippocampal networks work in a unified manner.

## 5 Discussion

Our main goal in this study was to evaluate the brain circuits underlying spatiotemporal memory processing under two levels of spatial similarity. Rats were able to discriminate the temporal order of object locations in both high and low interference conditions (i.e., highly similar locations and less similar locations). Although we did not detect different levels of c‐Fos expression in the regions of interest between the groups, we could see different patterns of functional network connectivity according to the level of spatial interference. Indeed, successful recall of spatiotemporal memory demanding higher levels of pattern separation seems to recruit a wider and more interconnected brain circuit.

The behavioral findings of the current study corroborate those reported on Hunsaker et al. (2008) since our rats were also able to discriminate object locations in different degrees of spatial similarity. It is important to note some differences between our protocol and the former: rat's strain and the spatial distances used. First, we have used Wistar rats instead of Long‐Evans, and despite their lower visual acuity (Prusky et al. 2002), the animals still have a similar performance compared to Long‐Evans rats. Second, since our open field has 90 cm of diameter, we could not use the distances applied by Hunsaker and Kesner, that is, 108 cm for low and 54 cm for high spatial interference (Hunsaker et al. 2008). Therefore, we have reduced the distances to 82.5 and 37.5 cm for LI and HI, respectively. Again, this modification did not affect the subjects' memory performance.

Although one could expect a lower performance of rats in the HI test condition, the discrimination ratio was similar between HI and LI groups, indicating that animals were able to disambiguate the displacement of objects positioned 37.5 cm apart (HI condition). Moreover, the total time of object exploration in the test sessions was not different between the groups, suggesting that both HI and LI animals had similar levels of motivation and memory performance; therefore, c‐Fos data could not be interpreted as the result of differences in exploration activity or discrimination ratio. Considering that c‐Fos protein peaks in the interval between 60 and 120 min after behavioral experience (Bisler et al. 2002; Barbosa et al. 2013), the IHC results in both LI and HI protocols may be related to the process of pattern separation in the sample phases and pattern completion in the test phase. Interestingly, HI and LI groups did not differ in the levels of c‐Fos expression in any analyzed brain regions. One likely explanation is due to the similarities between the behavioral protocols of HI and LI, which involve encoding and recalling temporal order memory for spatial locations. Olarte‐Sánchez et al. (2014) also did not find a difference in c‐Fos expression between experimental and control groups after the temporal order memory task with multiple trials. They also detected positive correlations among brain area activation by c‐Fos (PRH, LEC, DG, CA3, and CA1) and discrimination ratio in the experimental group. In this context, here we found a positive correlation between D2 and c‐Fos counts in the CA2 region, but only in the LI group. This result might suggest that behavioral performance is associated with CA2 neural activation in conditions of lower degrees of spatial similarity, consistent with the involvement of this area in pattern separation/completion (Lu et al. 2015) and temporal information processing (DeVito et al. 2009; Mankin et al. 2015). Given that this finding was not observed in the HI group, it suggests that the correlation depends on task contingencies, indicating the need for further studies to better understand this result.

Intriguingly, we have found stronger correlations in c‐Fos expression among the brain areas in HI when compared to the LI, suggesting that high interference conditions evoke a stronger coactivation of the network. More importantly, the HI group also presented networks with higher levels of local and global efficiency, which indicates a more robust and interconnected neuronal circuit in the condition that requires recall of information with high spatial interference. This result suggests that successful retrieval of temporal ordering for spatial locations demands functional connectivity of dorsal hippocampal regions with areas in the temporal lobe area (PRH35, PRH36, and DLENT), and that this network connectivity increases according to the levels of spatial pattern separation processing required by the task. Indeed, in the LI condition, we found that the network was organized in two loosely connected clusters, while in the HI condition, the Louvain method exhibited an integrated network organized in a single cluster. Similarly, Auguste et al. (2023) have also found a more interconnected neuronal circuit after rats recalled a what‐where‐when memory when compared to animals that recalled only the spatial component in a remote episodic task involving odors. In contrast to our findings, these authors showed a larger network recruited during recall, including a set of brain regions associated with odors‐linked emotional behavior. Future studies should also investigate the expression of other IEG across wider brain networks in order to have a global view of neuronal circuits underlying spatial pattern separation.

The main hubs formed by neuronal networks also differed between LI and HI conditions while in the LI we found that DG occupied a central spot; in the HI condition we found that CA1and DLENT organized the network. The role of the DG in spatial memory and pattern separation is well established (Hainmueller and Bartos 2020; Neves et al. 2008), and, from an anatomical perspective, the DG sends direct projections to CA3 and CA2, and receives back projections from CA3 (Senzai 2018), indicating that this area has a profound impact on hippocampal network functioning. Regarding the LI condition, where we have two clusters, it is worth mentioning that the DG is the only hippocampal subfield that coactivates with a parahippocampal area (i.e., DLENT), which might position this area as a main node from a functional connectivity perspective. In the HI condition, however, it is reasonable to assume a higher degree of difficulty due to similar spatial information across sample phases. There is plenty of evidence showing that CA1 participates in spatiotemporal processing (Kesner 2013; Hoge and Kesner 2007), and our findings show that CA1 activity covaries with all other areas while supposedly engaged in these computations. Considering that HI has a higher object‐location demand, this should also explain why DLENT became a hub in the neuronal circuit. Many studies show the involvement of the DLENT in the processing of object‐location tasks; for example, Kuruvilla et al. (2020) showed that lateral entorhinal cortex lesions lead to impairment in egocentric and allocentric versions of an object‐location recognition memory protocol, but not in the spatial T‐maze, indicating that this area is involved in an associative task. It is also interesting to note that PRH35 and PRH36 increased their apparent role in the network when the HI and LI conditions are compared. PRH35 and 36 are coactivated in the LI, and PRH36 and DLENT also have a positive correlation in the LI. On the other hand, both perirhinal regions increased their connectivity in the condition with higher spatial interference. The perirhinal cortex has strong structural connections with the lateral portion of the entorhinal cortex, but PRH36 also has bidirectional projections to CA1 (Kealy and Commins 2011). Our results suggest that the PRH can also be recruited in tasks with high spatial demands, which is in agreement with a theoretical proposal that the PRH‐CA1 circuit processes detailed sensory information (Burke et al. 2018).

Our study did not assess causal relations between the brain areas activated and memory performance, which should be investigated in future studies. However, the functional network connectivity analysis employed here is a powerful tool to provide a systemic view of the neuronal circuits underlying different cognitive and emotional processes. In future studies, it will be relevant to verify how network connectivity between these and other brain areas is necessary to recall spatiotemporal memory. For this purpose, it would be very important to use recent tools to evaluate causal relationships, such as optogenetics and chemogenetics. Furthermore, since IEGs show a limited temporal resolution, it would also be interesting to use electrophysiological methods to understand how some of these brain areas are processing spatiotemporal information across the different stages of memory formation and recall.

In conclusion, we detected the formation of different neuronal networks when rats properly recalled a spatiotemporal memory with different levels of spatial similarity. Higher spatial interference conditions lead to a wider and more interconnected functional brain network compared to lower spatial interference. Since pattern separation is crucial to episodic memory, our study advances understanding of this important memory system.

## Supporting information


Data S1.

## Data Availability

The data that support the findings of this study are available from the corresponding author upon reasonable request.
