# Supplementary material for: Brain Networks Differ According to Levels of Interference in Spatiotemporal Processing
Source: Hippocampus. 2025 Mar 24;35(2):e70011. doi: 10.1002/hipo.70011 (PMC11931270; doi:10.1002/hipo.70011)
Supplement: Supplementary file 1 — Data S1. [file HIPO-35-0-s001.docx]

**Supplementary Data**

Table 1 **-** Output values for Shapiro-Wilk’s test for normality on discrimination index for both conditions.

| Condition | Statistics | df | Sig. |
| --- | --- | --- | --- |
| HI | .986 | 8 | .988 |
| LI | .829 | 8 | .058 |

Table 2 **-** Output values for one-sample t-test against chance (0) for the discrimination indexes for both conditions.

| Condition | t | df | Sig. |
| --- | --- | --- | --- |
| HI | 7.323 | 7 | <.001 |
| LI | 5.078 | 7 | .001 |

Table 3 **-** Value of comparison statistics for c-Fos expression between groups.

| Area | U | p | Average post  (HI-LI) |
| --- | --- | --- | --- |
| PRh-36 | 27 | 0,64 | 7,88-9,13 |
| PRh-35 | 29 | 0,79 | 8,13-8,88 |
| DLENT | 27 | 0,64 | 7,88-9,13 |
| CA1 | 24 | 0,44 | 7,50-9,50 |
| CA2 | 31 | 0,95 | 8,38-8,63 |
| CA3 | 29 | 0,79 | 8,13-8,88 |
| DG | 31 | 0,75 | 8,63-8,38 |

Table 4 **-** Correlations between c-Fos and D2.

| Condition | Area | rho | p |
| --- | --- | --- | --- |
| HI | PRh-36 | 0,619 | 0,115 |
|  | PRh-35 | 0,238 | 0,582 |
|  | DLENT | 0,548 | 0,171 |
|  | CA1 | 0,619 | 0,115 |
|  | CA2 | 0,643 | 0,096 |
|  | CA3 | 0,405 | 0,327 |
|  | DG | 0,667 | 0,083 |
| LI | PRh-36 | 0,190 | 0,665 |
|  | PRh-35 | 0,286 | 0,501 |
|  | DLENT | 0,310 | 0,462 |
|  | CA1 | 0,714 | 0,058 |
|  | CA2 | 0,786 | **0,028*** |
|  | CA3 | 0,524 | 0,197 |
|  | DG | 0,452 | 0,268 |

*significant values ​​above chance.

Table 5 **-** Original values ​​of centrality measures and p value (bootstrap mean). Hubs were defined using the values ​​of the centrality measures: degree, betweenness, and strength, respectively. Firstly, we chose the highest values regarding the three measures, then we selected only the brain areas in which those measures were statistically significant.

|  | HI | | | LI | | |
| --- | --- | --- | --- | --- | --- | --- |
|  | Degree | Strength | Betweeness | Degree | Strength | Betweeness |
| PRh-36 | **5*(4.31)** | **4.38* (3.07)** | 0.66(0.56) | **2*(2.23)** | 1.69(2.39) | **5* (2.31)** |
| PRh-35 | **3*(4.22)** | **2.25*(3.64)** | **0*(0.53)** | **1*(1.8)** | **0.74* (2.15)** | **0*(1.30)** |
| DLENT | **6*(4.8)** | **5.33* (4.14)** | **1.83*(0.92)** | **2*(2.08)** | 1.69(2.48) | **8*(1.72)** |
| DG | **3*(4.25)** | **2.54* (3.67)** | **0*(0.5)** | **3*(2.7)** | **2.40* (2.56)** | **9*(2.99)** |
| CA3 | **4*(4.6)** | **3.33* (3.96)** | **0*(0.69)** | **2*(2.2)** | **1.55* (2.60)** | **0*(2.26)** |
| CA2 | **3*(4.73)** | **4.35* (4.08)** | **0.66*(0.74)** | **3*(2.48)** | **2.50* (2.88)** | 2(2.44) |
| CA1 | **6*(5.09)** | **5.22* (4.38)** | **1.83*(1.04)** | **3*(2.51)** | **2.61* (2.52)** | 2(2.30) |

*significant values ​​above chance.

Table 6 **-** Efficiency measures value and bootstrap mean.

| Measures | Global Efficiency | Cluster Coefficient |
| --- | --- | --- |
| Condition | Value (Bootstrap Mean) | Value (Bootstrap Mean) |
| HI | 1.03 (1.03) | **0.74* (0.76)** |
| LI | **0.79* (0.83)** | 0.49 (0.50) |

*significant values ​​above chance.

Table 7 **-** Comparison of network efficiency measures.

| Measure | p | Wilcox value | median LI | median HI |
| --- | --- | --- | --- | --- |
| Global Efficiency | **0,001*** | 1000 | 0,79 | 1,03 |
| Cluster Coefficient | **0,001*** | 1000 | 0,49 | 1,03 |

*significant values ​​above chance.

Table 8 **-** Lovain cluster analysis values, modules and modularity

| Condition | (Number of modules) areas | Modularity value ( Bootstrap Mean) |
| --- | --- | --- |
| HI | (1) PRH-36, PRH-35, DELENT, DG, CA3,CA2,CA1 | **0,13 (0.10)*** |
| LI | (1) PRH-36, PRH-35, DELENT,  (2) DG, CA3,CA2, CA1 | **0,37 (0.23)*** |

*significant values ​​above chance
